# Stn1 is critical for telomere maintenance and long-term viability of somatic human cells

**DOI:** 10.1111/acel.12289

**Published:** 2015-02-14

**Authors:** Virginia Boccardi, Neetu Razdan, Jessica Kaplunov, Jyoti J Mundra, Masayuki Kimura, Abraham Aviv, Utz Herbig

**Affiliations:** 1 Department of Geriatric Medicine and Metabolic Diseases, Second University of NaplesNaples, Italy; 2 Department of Microbiology, Biochemistry & Molecular Genetics, Rutgers Biomedical and Health SciencesNewark, NJ, USA; 3 Department of Biochemistry and Molecular Biology, Rutgers Biomedical and Health SciencesNewark, NJ, USA; 4 Center of Human Development and Aging, Rutgers Biomedical and Health SciencesNewark, NJ, USA; 5 New Jersey Medical School-Cancer Center, Rutgers Biomedical and Health SciencesNewark, NJ, USA

**Keywords:** cellular senescence, CST, oxidative stress, Stn1, telomere dysfunction, telomere erosion

## Abstract

Disruption of telomere maintenance pathways leads to accelerated entry into cellular senescence, a stable proliferative arrest that promotes aging-associated disorders in some mammals. The budding yeast CST complex, comprising Cdc13, Stn1, and Ctc1, is critical for telomere replication, length regulation, and end protection. Although mammalian homologues of CST have been identified recently, their role and function for telomere maintenance in normal somatic human cells are still incompletely understood. Here, we characterize the function of human Stn1 in cultured human fibroblasts and demonstrate its critical role in telomere replication, length regulation, and function. In the absence of high telomerase activity, shRNA-mediated knockdown of hStn1 resulted in aberrant and fragile telomeric structures, stochastic telomere attrition, increased telomere erosion rates, telomere dysfunction, and consequently accelerated entry into cellular senescence. Oxidative stress augmented the defects caused by Stn1 knockdown leading to almost immediate cessation of cell proliferation. In contrast, overexpression of hTERT suppressed some of the defects caused by hStn1 knockdown suggesting that telomerase can partially compensate for hStn1 loss. Our findings reveal a critical role for human Stn1 in telomere length maintenance and function, supporting the model that efficient replication of telomeric repeats is critical for long-term viability of normal somatic mammalian cells.

## Introduction

Telomeres are evolutionarily conserved nucleoprotein structures at the ends of linear chromosomes. Their primary function is to prevent the cell from sensing chromosome ends as breaks in double-stranded DNA, thereby suppressing the activation of a DNA damage response (DDR). In humans, telomeres consist of repetitive TTAGG sequences that are associated with shelterin, a six subunit protein complex (Palm & de Lange, 2008), among others. Telomere lengths, however, are not static. With every division of somatic cells in culture, and presumably *in vivo,* telomeres progressively shorten by 50–200 bp until one or few telomeres become dysfunctional. The ensuing telomeric DDR typically leads to a permanent proliferative arrest termed cellular senescence or telomere dysfunction-induced cellular senescence (TDIS).

As telomeres progressively erode with every cell division, they are thought to function as replicative timers that initiate a growth arrest once a critical length is reached (Harley *et al*., 1990). Factors that contribute to progressive telomere erosion include the inability of the replicative polymerase to completely duplicate linear chromosomes (the end replication problem), nucleolytic processing of telomeric ends, and sporadic telomere attrition due to defective telomere maintenance and repair mechanisms. In addition, cell extrinsic stresses, such as reactive oxygen species (ROS), can accelerate the rate at which cells enter TDIS, although the reasons for ROS-induced telomere dysfunction are poorly understood. Furthermore, due to their repetitive nature and tendency to form secondary structures, telomeres also pose a challenge to the DNA replication machinery. This raises the possibility that yet another reason for progressive telomere erosion observed with every cell division is due to premature termination of telomeric DNA synthesis.

Telomerase, a ribonucleoprotein complex, can prevent progressive telomere erosion by adding TTAGGG repeats to the ends of G-rich telomeric strands. Synthesis of the complementary C-rich strand is accomplished by other factors. In budding yeast, a trimeric protein complex composed of Cdc13, Stn1, and Ten1 (CST) plays a critical role in both telomere replication and telomere end protection (Price *et al*., 2010). CST binds to G-rich telomeric single-stranded DNA (ssDNA) and coordinates C-strand synthesis by directly interacting with and modulating DNA polymerase α activity. It also regulates extension of the G-rich telomeric strand by coordinating recruitment of telomerase to the chromosome ends. CST, consisting of Ctc1, Stn1, and Ten1, has recently been identified in mammals, where its role in regulating telomere lengths and function is slowly emerging.

Mammalian CST is thought to be involved primarily in the replication of telomeric repeats, although nontelomeric functions have also been suggested (Price *et al*., 2010). CST has both structural and functional homology to the DNA replication protein complex RPA (Miyake *et al*., 2009); it binds to single-stranded DNA (Miyake *et al*., 2009) and directly interacts with and stimulates the activity of DNA polymerase α (Casteel *et al*., 2009). Knockdown of either Stn1 or Ctc1 in human cancer cells and in mouse fibroblasts results in substantially elongated telomeric G-overhangs, consistent with its recently demonstrated role in facilitating telomeric C-strand synthesis and negatively regulating telomerase (Surovtseva *et al*., 2009; Dai *et al*., 2010; Chen *et al*., 2012; Wang *et al*., 2012; Wu *et al*., 2012). Conditional deletion of *CTC1* from mice leads to rapid and catastrophic telomere attrition, early entry into senescence, and signs of telomeric replication defects, such as fragile telomeres and inefficient restart of stalled telomeric replication forks (Gu *et al*., 2012). These telomeric replication defects were also observed in human cancer cells subjected to shRNA-mediated Stn1 knockdown, although telomere lengths and cellular proliferation rates did not appear to be affected (Dai *et al*., 2010; Stewart *et al*., 2012; Wu *et al*., 2012). Given that most cancer cells lines express high levels of telomerase, it is possible that reduced levels of Stn1 are inconsequential for the viability of cells in which telomere lengths can be maintained efficiently. Thus, whether Stn1 function is equally important for telomere replication and cell viability in normal mammalian cells is still poorly understood.

In this study, we characterized the effects of Stn1 knockdown on telomere structure, function, and cell viability in normal human fibroblasts and in fibroblasts expressing the catalytic subunit of telomerase, hTERT. We report that loss of Stn1 function, regardless of hTERT expression status, results in telomere replication defects. However, in cells lacking high telomerase activity, these defects cause stochastic telomere attrition, accelerated telomere erosion rates, telomere dysfunction, and consequently early entry into senescence. In contrast, hTERT-expressing cells did not display any detectable defects in cell cycle progression or cell viability, demonstrating that telomerase can counteract telomeric replication defects due to loss of Stn1 function. We also observed that in the absence of high telomerase activity, oxidative stress dramatically accelerates telomeric defects caused by Stn1 depletion, indicating an important role for Stn1 in replication telomeres damaged by oxidative stress.

## Results

### Knockdown of Stn1 reduces lifespan of normal human fibroblasts in a dose-dependent manner

**Fig 1:**
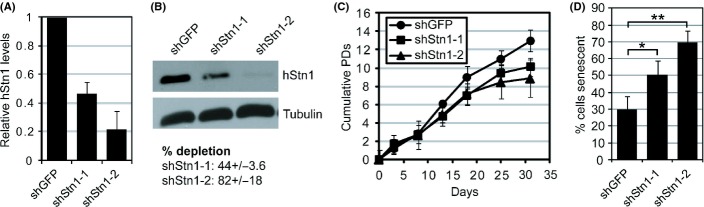
Knockdown of hStn1 causes accelerated entry into cellular senescence in normal human cells. Levels of hStn1 knockdown were determined after cells had recovered from drug selection, for 48 h. (A) mRNA levels of hStn1, as measured by quantitative RT–PCR, in BJ cells stably expressing shRNAs directed against two distinct sequences in hStn1 (shStn1-1 and shStn1-2) and against GFP, as a control. (B) Western blot of total protein extracts from BJ cells stably expressing indicated shRNAs. Numbers below the blot indicate the percentage of hStn1 knockdown as measured by densitometry, compared to GFP controls, from three distinct experiments. One representative Western blot is shown. (C) Proliferation curves of BJ cells stably expressing indicated shRNAs. Values are expressed as mean ± standard deviation (SD) from three independent experiments performed in duplicate. The cumulative number of population doublings (PDs) was assessed at each passage. (D) Quantitation of the percentage of senescent cells, as measured by the lack of BrdU incorporation added to the culture medium for 48 h, 4 weeks following retroviral transduction. Data are expressed as mean ± SD from three independent experiments; *P = 0.036 **P = 0.002.

Yeast Stn1 is critical for both chromosome end protection and replication of telomeric repeats. Whether Stn1 plays similar roles in normal somatic human cells, however, is less clear. To test the effects of Stn1 depletion on telomere structure, function, and cell viability of normal human cells, we stably expressed shRNA against two distinct sequences (Casteel *et al*., 2009) in the coding region of Stn1 (termed shStn1-1 and shStn1-2) in BJ (ATCC) fibroblasts using retroviral transduction followed by drug selection. As a control, BJ cells expressing shRNA against a sequence of green fluorescence protein (shGFP) were also generated. Efficiency of Stn1 knockdown was evaluated by RT–PCR, revealing an average reduction in Stn1 mRNA levels by 54% and 79% in shStn1-1 and hStn1-2 cells, respectively (Fig.1A). Similarly, immunoblotting using anti-Stn1 antibodies revealed a reduction in Stn1 protein levels by 44% for shStn1-1-expressing cells and 82% for shStn1-2 cultures, when compared to shGFP cells (Fig.1B). Thus, while shStn1-1 was able to moderately reduce expression of hStn1 in normal human fibroblasts, stable expression of shStn1-2 resulted in an efficient knockdown of approximately 80% on both the mRNA and protein levels.

To determine whether hStn1 is essential for cell viability of normal human fibroblasts, we serially passaged hStn1 knockdown and control cells in culture under physiologic 2% oxygen conditions until cells entered senescence. While cell growth kinetics of all three cultures were similar early after retroviral transduction, a decrease in growth rates was observed in hStn1 knockdown cells compared to control cells, 8 days after recovery from drug selection (Fig.1C). In contrast to shGFP cultures that underwent cellular senescence at population doubling (PD) 25, shStn1-1- and shStn1-2-expressing cultures ceased proliferating already at PD 10 and 8, respectively (Fig.1C and data not shown). The senescence status was verified by staining for senescence-associated β-galactosidase (SA-β-gal) activity, by the appearance of a characteristically enlarged and flattened morphology (Fig. S1, Supporting information), and by the lack of BrdU incorporation, added to the culture medium for 48 h (Fig.1D and Fig. S1, Supporting information). Significantly less replicative capacity was consistently observed in cultures in which hStn1 knockdown was most efficient (shStn1-2), indicating that it is tightly correlated with hStn1 levels and function (Fig.1C). Our data demonstrate that hStn1 is essential for long-term viability of normal human fibroblasts and that reducing hStn1 levels dramatically shortens their replicative capacity.

### Early entry into senescence of hStn1 knockdown cells is a result of telomere dysfunction

Replicative exhaustion of human skin fibroblasts is a result of progressive telomere erosion and dysfunction. Dysfunctional telomeres are sensed as double-stranded DNA breaks (DSBs) and consequently associate with a number of DDR factors such as γH2AX and p53BP1. The accumulation of these telomeric DDR factors then signals the activation of a permanent DDR and initiates cellular senescence (d'Adda di Fagagna *et al*., 2003; Gire *et al*., 2004; Herbig *et al*., 2004).

**Fig 2:**
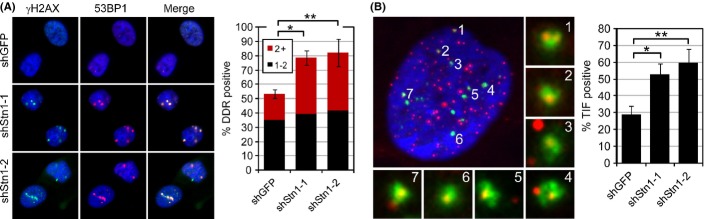
Knockdown of hStn1 causes telomere dysfunction in normal human cells. (A) BJ cells transduced with indicated retroviral combinations were immunostained with antibodies against γH2AX (green) and 53BP1 (red) 4 weeks following transduction. Nuclei were counterstained with DAPI (blue). Quantitation of DDR-positive cells, defined as a cell nucleus with one (black bars) or two and greater (red bars) colocalizations between γH2AX and 53BP1. At least 100 cells were analyzed for each group; *P = 0.001 **P = 0.007. (B) Dysfunctional telomeres in hStn1 knockdown cells. Cells were processed by immunoFISH to simultaneously detect 53BP1 (green) and telomeres (red). Nuclear DNA was counterstained with DAPI (blue). Enlarged versions of the numbered DDR foci showing colocalization with telomeres are shown in the right and below. Bar graph: quantitation of TIF-positive cells in indicated samples (mean ± SD; *n* = 3). A cell was considered TIF positive when ≥ 50% of 53BP1 foci colocalized with telomeres in a given cell nucleus. At least 50 cells were analyzed for each group; *P = 0.007 **P = 0.005.

To determine whether accelerated entry into senescence in shStn1 cells was a consequence of telomere dysfunction, we immunostained cells with antibodies against γH2AX and p53BP1, and analyzed DDR focus formation by immunofluorescence microscopy. While only ∼52% of shGFP cells were positive for DDR foci at the time of analysis, ∼79% of cells in near-senescent hStn1 knockdown cultures displayed discrete DDR foci (Fig.2A). Of note, the increase in DDR foci was statistically significant only in cells with multiple foci. To determine whether the observed DDR was a consequence of telomere dysfunction, we simultaneously detected DDR foci using antibodies against 53BP1 and labeled telomeres using a Cy3-conjugated telomeric peptide nucleic acid (PNA) and characterized presence of telomere dysfunction-induced DNA damage foci (TIF; (Takai *et al*., 2003)) by ApoTome microscopy (Suram *et al*., 2012). We discovered that most cells in hStn1 knockdown cultures were TIF positive, defined as a cell in which at least 50% of 53BP1 foci colocalized with telomeres (Fig.2B). Our data therefore demonstrate that the reduced replicative capacity of Stn1 knockdown cultures is a consequence of telomere dysfunction.

### hStn1 deletion in normal human fibroblasts leads to fragile telomeres and accelerated telomere erosion

**Fig 3:**
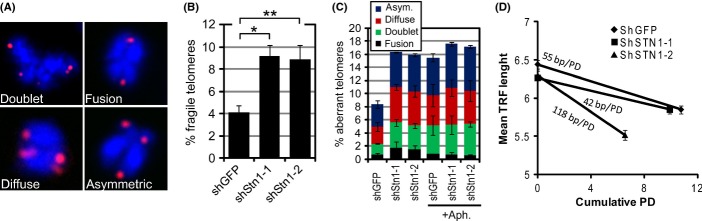
Knockdown of hStn1 causes fragile telomeres and accelerated telomere erosion rates. (A) Metaphase chromosomes (blue) were processed by FISH using a telomeric peptide nucleic acid probe (red) 1 week following hStn1 knockdown. Representative images of observed aberrant telomeric structures are shown. (B) Quantification of fragile telomeres, defined as diffuse or multitelomeric signals on a single chromatid, in indicated knockdown cells. Mean values from three independent experiments are shown (± SD). At least 4000 telomeres were analyzed for each group and each experiment; *P = 0.024; **P = 0.041. (C) Quantitation of aberrant telomeres in indicated cultures in the absence and presence of 0.2 μm aphidicolin; shGFP vs. shGFP-Aph *P = 0.0485; shGFP vs. shStn1-1 *P* = 0.028; shGFP vs. shStn1-2 *P* = 0.002. (D) telomere erosion rates measured using TRF analysis. Values are expressed as mean ± SD from a total of two independent experiments performed in duplicate.

Although the exact function of human Stn1 is still unclear, it has been suggested to play a role in facilitating replication of chromosomal DNA, including telomeric repeats (Casteel *et al*., 2009; Price *et al*., 2010). As knockdown of hStn1 accelerated the rate at which telomeres became dysfunctional, but did not trigger immediate telomere dysfunction, it was possible that reducing levels of Stn1 prevents efficient replication of telomeres, thereby accelerating telomere erosion and dysfunction. To test this possibility, we analyzed cells for the presence of fragile telomeres, a hallmark of telomeric replication defects (Martinez *et al*., 2009; Sfeir *et al*., 2009). Fragile telomeres can be detected on metaphase chromosomes by telomere FISH and are characterized by the presence of multiple or diffuse telomeric signals on a single chromatid arm. Cells depleted of shStn1 indeed displayed a greater than twofold increase in fragile telomeres compared to control cells, suggesting that Stn1 knockdown impedes replication of telomeric repeats (Fig.3A,B). In addition, we also observed other aberrant telomeric structures in shStn1 knockdown cultures such as fusions of sister telomeres, dramatically different intensities of sister telomere FISH signals, and telomere signal-free ends (Fig.3A). Together, these aberrant telomeric structures were observed twofold more frequently in hStn1 knockdown cells compared to control cells (Fig.3C). No other chromosomal abnormalities such as end-to-end fusions or nontelomeric chromosome breaks were apparent in analyzed metaphases. Furthermore, the drug aphidicolin, an inhibitor of polymerase α which by itself causes an increase in fragile telomeres (Sfeir *et al*., 2009), did not additionally increase the fragile and aberrant telomere phenotype of hStn1 knockdown cultures. These data suggest that hStn1 and DNA polymerase α act in the same pathway to suppress replication defects in telomeric repeats.

One of the most noticeable telomeric aberrations caused by hStn1 knockdown was the partial or complete loss of sister telomere signals (Fig.3B,C), suggesting that loss of hStn1 function leads to stochastic telomere attrition. To test this further, we analyzed terminal restriction fragment (TRF) lengths by Southern blotting and consistently observed shorter mean telomere lengths in near-senescent Stn1-2 knockdown cultures compared to control cells, analyzed 31 days following expression of the shRNA (Fig.3D and see below). We calculate a loss of 118 bp per PD for shStn1-2 cultures, more than double the telomere erosion rates of shGFP control cultures (55 bp/PD; Fig.1). Surprisingly, we did not detect significantly different telomere erosion rates in shStn1-1 knockdown cultures (42 bp/PD, Fig.3D) compared to control cells, suggesting that only partial knockdown of Stn1 does not affect telomere erosion rates to a degree that is detectable by TRF analysis. Given that Stn1 has at the least two distinct functions during telomere duplex replication, facilitating restart of stalled telomeric replication forks and participating in C-strand fill-in synthesis, it is possible that only one of these functions is disrupted by partial knockdown (Wang *et al*., 2012).

### The effects of hStn1 depletion are enhanced by oxidative stress

**Fig 4:**
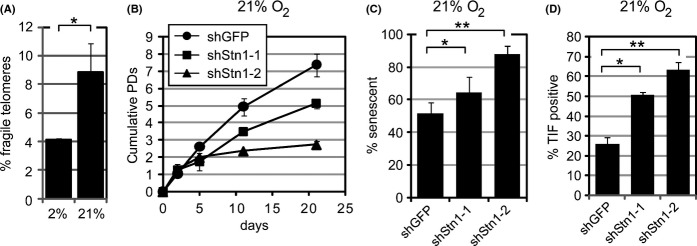
Atmospheric 21% oxygen tensions cause fragile telomeres and augment the effects of hStn1 depletion. (A) Quantitation of fragile telomeres of human BJ fibroblasts grown in a 2% and 21% oxygen atmosphere. *P = 0.070. (B) Proliferation curves of human fibroblasts grown in a 21% oxygen atmosphere and stably expressing indicated shRNAs. Values are expressed as mean ± standard deviation (SD) from two independent experiments in duplicate. (C) Quantification of the percentage of senescent cells 21 days following transduction of indicated retroviral shRNA constructs and grown in a 21% oxygen atmosphere. Error bars represent SD; *P = 0.048;**P = 0.026. (D) Quantification of TIF-positive cells in indicated cultures (mean ± SD; *n* = 2) that were grown in a 21% oxygen atmosphere. A cell was considered TIF positive when ≥ 50% of 53BP1 foci colocalized with telomeres in a given cell nucleus. At least 50 cells were analyzed for each group; *P = 0.012; **P = 0.009.

Oxidative stress causes DNA damage that impedes telomere replication and results in sister telomere loss when DNA damage repair is challenged (Wang *et al*., 2010). In agreement with these data, we discovered that culturing cells under 21% oxygen conditions also increased the percentage of fragile telomeres compared to more physiological 2% oxygen culture conditions (Fig.4A). Knockdown of hStn1 in a 21% oxygen atmosphere (Fig. S2A, Supporting information), or addition of aphidicolin, did not further increase the fragile telomere phenotype of human fibroblasts (Fig. S2B, Supporting information), supporting the model that Stn1 functions in the same pathway as polymerase α to facilitate replication of telomeres damaged by oxidative stress. However, as the effects of Stn1 knockdown on the cells' proliferative capacity were dramatically enhanced in a 21% oxygen atmosphere, our inability to observe a further increase in fragile telomeres might also be because most cells were senescent and consequently could not enter mitosis. While Stn1 knockdown cells entered senescence 8–10 population doublings following expression of the knockdown vector in 2% oxygen conditions (Fig.1C), hStn1-2 knockdown cells cultured in a 21% oxygen atmosphere entered senescence after a mere 2 population doublings, as detected by cell proliferation curves (Fig.4B) and BrdU incorporation assays (Fig.4C). Although we were unable to reliably measure telomere erosion rates of cultures grown under normoxic conditions, due to this rapid entry into senescence, permanent growth arrest was accompanied by the induction of a DDR (Fig. S2C, Supporting information) and the formation of dysfunctional telomeres (Fig.4D). Similar to cells cultured under hypoxic conditions, the effects of hStn1 knockdown were greatest in cultures that displayed the most efficient hStn1 knockdown, that is, cells expressing shStn1-2 (Fig. S2A, Supporting information). Thus, hStn1 suppresses extensive telomere loss and premature entry into TDIS, presumably by facilitating the replication of telomeres that had been damaged by oxidative stress.

To determine whether overexpression of Stn1 can suppress accelerated telomere erosion and dysfunction rates due to telomeric replication stress, we generated BJ cell cultures that stably overexpressed Stn1 from a retroviral promoter. Despite dramatically higher Stn1 expression levels compared to control cultures, these cells did not show signs of reduced telomeric replication stress and, similar to Stn1 knockdown cultures, entered TDIS at earlier population doublings compared to controls (Fig. S3A–F, Supporting information). Furthermore, cells overexpressing Stn1 were not resistant to ROS- and drug-induced DNA replication stress and showed telomeric defects similar to control cultures (Fig. S3G,Supporting information. As Stn1 functions in a complex together with Ctc1 and Ten1, it is likely that higher than normal levels of Stn1 alone do not facilitate replication of telomeric repeats, rather, they might even interfere with the function of endogenous CST thereby reducing overall replicative capacity.

### The effects of hStn1 depletion in telomerized human fibroblasts

**Fig 5:**
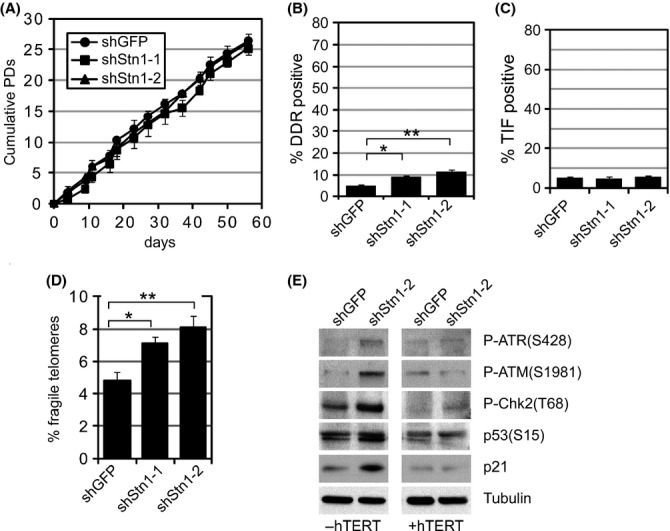
Knockdown of hStn1 in telomerized human fibroblasts causes a fragile telomere phenotype but does not lead to telomere dysfunction or to proliferation defects. (A) Proliferation curves of hTERT-expressing human fibroblasts stably expressing indicated shRNA constructs. Values are expressed as mean ± standard deviation (SD) from three independent experiments. The effect of hStn1 knockdown was monitored after the cells had recovered from transduction in fresh media for 48 h (indicated as day 0). (B) Quantification of DDR-positive cells, defined as a cell nucleus with at least one colocalization between γH2AX and 53BP1, 4 weeks following transduction of indicated shRNA. At least 100 cells were quantified for each group; *P = 0.0245;**P = 0.0136. (C) Quantitation of colocalizations between 53BP1 and telomeres in indicated samples 4 weeks following retroviral transduction (mean ± SD; *n* = 3). A cell was considered TIF positive when ≥ 50% of 53BP1 foci colocalized with telomeres in a given cell nucleus. At least 50 cells were analyzed for each group; No significant differences could be established. (D) Quantification of fragile telomeres, defined as telomeric doublets and diffuse telomeric signals on metaphase chromosomes, in indicated cells. Bars represent mean values of two independent experiments with SDs. At least 4000 telomeres were analyzed for each group in each experiment; *P = 0.037; **P = 0.033. (E) Immunoblots using antibodies against indicated DNA damage checkpoint factors in control GFP knockdown cells (shGFP) and Stn1 knockdown cells (shStn1-2). β-Tubulin was used as loading control.

We recently demonstrated that hTERT expression can suppress the proliferative defects caused by oncogene- and drug-induced DNA replication stress in normal human somatic fibroblasts (Suram *et al*., 2012). To test whether hTERT can also compensate for loss of Stn1 expression, we generated a fibroblast cell strain that stably expresses the catalytic subunit of telomerase hTERT. Expression of Stn1 was subsequently suppressed using the shRNA constructs as described above, resulting in a similar reduction in Stn1 mRNA and protein levels compared to normal BJ cells (Fig. S4A, Supporting information). Despite a highly efficient knockdown, shStn1 cells did not display any significant growth defects and proliferated at similar rates compared to shGFP control cultures for at least 2 months, after which we ceased to analyze growth curves (Fig.5A). In addition, no reduction in total telomere lengths could be detected as a result of Stn1 knockdown in BJ-hTERT cells (not shown).

Consistent with observed normal growth kinetics, hTERT expression dramatically reduced the percentage of cells that stained positive for DDR factors 53BP1 and γH2AX in both control and Stn1 knockdown cultures (Fig.5B). Despite this reduced DDR, however, Stn1 knockdown resulted in a statistically significant increase in DDR-positive cells (Fig.5B). The great majority of these DDR foci in these cells, however, did not colocalize with telomeric repeats making most cells TIF negative (Fig5C). Similar results were obtained from cells grown in 21% oxygen conditions (not shown). As Stn1 knockdown also resulted in an increase in chromosomal aberrations, such as an increase in fragile sites detected on metaphase chromosomes, it is likely that these nontelomeric DDR foci were a result of chromosome breakage at nontelomeric loci (Fig. S4B, Supporting information). Despite the lack of dysfunctional telomeres, however, expression of hTERT did not rescue the fragile telomere phenotype of hStn1 knockdown cultures. One week following drug selection, ∼8% of telomeres in shStn1 cells showed aberrant and fragile structures, a significant increase compared to control cells (Fig.5D). Similar to normal BJ cells, aphidicolin did not significantly increase the fragile telomere phenotype due to hStn1 knockdown (Fig. S4C, Supporting information). Furthermore, although we did not detect erosion of total telomere lengths upon hStn1 knockdown, we did observe a modest, yet significant increase in sister telomere loss in hTERT-expressing cells (Fig. S4D, Supporting information).

Our data demonstrate that hTERT expression rescues formation of dysfunctional telomeres and cellular senescence following Stn1 knockdown. However, as hTERT expression did not suppress the fragile telomere phenotype in Stn1 knockdown cultures, our data also suggest that formation of fragile telomeres is insufficient to trigger a DNA damage checkpoint and growth arrest. This is consistent with a number of studies that have demonstrated the presence of fragile telomeres in proliferating cells with high telomerase activity, such as cancer cells. To test this prediction directly, we analyzed DNA damage checkpoint activation following Stn1 knockdown in normal human fibroblasts that either lacked or overexpressed hTERT. Immunoblotting against ATR(S428), ATM(S1981), Chk2(T68), p53(S15), and p21 revealed strong activation of a DNA damage checkpoint in normal human fibroblasts depleted of Stn1, but not in Stn1 knockdown fibroblasts overexpressing hTERT. Thus, while it cannot suppress the fragile telomere phenotype, hTERT rescues telomere dysfunction, DNA damage checkpoint activation, and cellular senescence of somatic human cells in response to Stn1 depletion.

## Discussion

Mammalian CST plays important and multiple roles during telomere replication and telomere length regulation. Ctc1, a member of the CST complex, is evidently essential for efficient telomere replication by facilitating restart of stalled DNA replication forks. Its deletion in mouse embryonic fibroblasts prompted catastrophic and stochastic telomere loss, followed by rapid entry into cellular senescence after two population doublings (Gu *et al*., 2012). Our study demonstrates a critical role for Stn1 in regulating telomere lengths and replicative potential of normal human fibroblasts. Although knockdown of Stn1 in normal somatic cells at 2% oxygen tensions did not cause immediate cessation of cell proliferation, it decreased their proliferative capacity substantially. Stn1 knockdown cells displayed a greater than twofold increase in telomere erosion rates over control cultures and entered cellular senescence as a consequence of telomere dysfunction. In addition, suppression of Stn1 expression resulted in a significant increase in sister telomere loss and in the formation of fragile telomeres, a hallmark of telomeric replication stress (Martinez *et al*., 2009; Sfeir *et al*., 2009). Accordingly, the reduced proliferative capacity of Stn1 knockdown cultures was likely due to their inefficient replication of telomeric sequences, leading to accelerated telomere erosion rates and accelerated entry into TDIS.

Our data and those from a previous study in which knockout of CTC1 resulted in similar telomeric and proliferative defects in mouse embryonic fibroblasts (Gu *et al*., 2012) may seem at odds with studies demonstrating that knockdown of either Stn1 or Ctc1 did not result in telomere dysfunction or any significant growth defects in human cancer cell lines such as HeLa (Casteel *et al*., 2008; Miyake *et al*., 2009; Surovtseva *et al*., 2009) and HT1080 (Chen *et al*., 2012). However, HeLa cells and most other cancer cell lines display high levels of telomerase which might compensate for some of the defects caused due to partial loss of CST expression. One recently discovered function of human CST is to negatively regulate excessive telomere extension by telomerase in S/G2 phase of the cell cycle (Chen *et al*., 2012). Consequently, knockdown of human Stn1 and Ctc1 in cells with high telomerase activity resulted in progressive and telomerase-dependent telomere length extension. This finding contrasts with telomere attrition that is observed in human cells with undetectable telomerase activity (our data) and in mouse cells (Gu *et al*., 2012) that display significantly lower telomerase activity compared to cancer cells. Our data therefore provide important insights into the differences in telomere length maintenance between cells expressing high levels of telomerase, such as cancer cells, and normal mammalian somatic cells.

How could high levels of telomerase compensate for partial loss of CST expression? Suppressing expression of mammalian Stn1 and Ctc1 leads to telomeric replication defects, as evident by the formation of fragile telomeres, shown here and by others (Gu *et al*., 2012). However, hTERT overexpression could not suppress the formation of fragile telomeres generated as a result of Stn1 knockdown (Fig.5D), suggesting that telomerase is unable to suppress telomeric replication stress. This is in agreement with data demonstrating that high telomerase levels cannot prevent formation of fragile telomeres generated as a result of drug- or oncogene-induced DNA replication stress (Sfeir *et al*., 2009; Suram *et al*., 2012). Although hTERT-expressing Stn1 knockdown cells still encountered a modest increase in sister telomere loss compared to control cells (Fig. S3C, Supporting information), these sporadic telomere attrition events did not induce detectable changes to cell proliferation kinetics or to loss of cell viability (Fig.5A). Thus, despite its inability to suppress stalling of telomeric replication forks, telomerase can ostensibly compensate for sporadic telomere attrition and dysfunction, which is in agreement with our previous data (Suram *et al*., 2012). Precisely, how telomerase can suppress the formation of dysfunctional telomeres in response to Stn1 knockdown, or DNA replication stress, is unclear. One possibility is that telomerase synthesizes new TTAGGG repeats at lesions generated as a consequence of telomeric replication fork stalling by a process that resembles, or is identical to chromosome healing (Murnane, 2010), thereby suppressing the activation of a telomeric DDR.

Mammalian cells cultured in 21% oxygen enter replicative senescence with longer telomeres and at earlier population doublings compared to cells grown in the presence of antioxidants or in 2% oxygen atmosphere (Chen *et al*., 1995; von Zglinicki *et al*., 2000; Betts *et al*., 2008; Britt-Compton *et al*., 2009). The cause for skin fibroblast senescence in either oxygen tension is telomere dysfunction (d'Adda di Fagagna *et al*., 2003; Gire *et al*., 2004; Herbig *et al*., 2004). One interpretation of these data is that free radicals and peroxides cause stochastic telomere attrition and dysfunction while leaving most telomeres long and functional. The G-rich nature of mammalian telomeres makes these repetitive sequences particularly prone to oxidative damage. Oxidized guanines in telomeric repeats interfere with binding of the shelterin components TRF1 and TRF2 (Opresko *et al*., 2005), which could either directly interfere with telomere capping, or alternatively, impede telomere replication as TRF1 is critical for this process (Sfeir *et al*., 2009). Furthermore, telomere lagging strand synthesis is defective in cells devoid of Ogg1, a DNA glycosylase that is critical for the repair of telomeric 8-oxo-dG, lesions generated by ROS (Wang *et al*., 2010). In addition, cells subjected to H2O2 or 40% oxygen display an increase in single-stranded DNA nicks in telomeric repeats, lesions that are repaired inefficiently and evidently interfere with replication fork progression (Petersen *et al*., 1998; von Zglinicki *et al*., 2000). We now provide additional evidence that oxidative stress leads to defective telomere replication by demonstrating that 21% oxygen culture conditions caused fragile telomeres. This finding is in line with the notion that telomeric replication stress causes stochastic telomere attrition, telomere dysfunction, and consequently cellular senescence in normal human fibroblasts (Suram *et al*., 2012). Our data presented here therefore suggest that accelerated entry into replicative senescence of cells cultured in 21% oxygen is a consequence of oxidative stress-induced telomeric replication defects.

Knockdown of Stn1 further enhanced the proliferative defects of normal fibroblasts cultured in 21% oxygen and cells underwent TDIS after just two population doublings. Elevated oxygen tensions increase the levels of intracellular ROS, and ROS generate lesions in telomeric repeats that potentially cause replication fork stalling. We thus propose that Stn1 facilitates replication of telomeric sequences damaged by ROS. Similar to Ctc1, this function of Stn1 potentially involves facilitating restart of replication forks stalled at telomeric lesions (Stewart *et al*., 2012). Failure to restart telomeric replication forks, due to Ctc1 loss, has been shown to lead to an increase in fragile telomeres, stochastic and accelerated telomere attrition, an increase in single-strand G-rich telomeric DNA, and a dramatically shortened replicative potential (Gu *et al*., 2012). These changes to telomere structure and cell viability were also observed in response to Stn1 knockdown (our data and Miyake *et al*., 2009; Wu *et al*., 2012), supporting the model that Stn1 and Ctc1 function as a complex to facilitate telomere replication in mammals.

Increasing evidence suggests a causative role for cellular senescence in promoting aging and aging-related diseases in mammals. Cells with features of senescence accumulate in various tissues of aging mice (Krishnamurthy *et al*., 2004; Hewitt *et al*., 2012), nonhuman primates (Herbig & Sedivy, 2006; Jeyapalan *et al*., 2007; Fumagalli *et al*., 2012), and humans (Dimri *et al*., 1995; Suram *et al*., 2012), and preventing the accumulation of senescent cells has been demonstrated to significantly improve healthspan in mice (Baker *et al*., 2011). As mammals get older, telomeres progressively become shorter in many of their replicative somatic tissues and therefore likely contribute to the accumulation of senescent cells with advancing age (Zhu *et al*., 2011). Shorter leukocyte telomere lengths (LTL) are associated with aging-associated disorders in humans, including cardiovascular disease (CVD), while healthy lifespan is positively correlated with longer telomeres (Aviv, 2012). Accordingly, changes leading to accelerated or reduced telomere erosion rates may to some extent facilitate or suppress the formation of some aging-associated disorders, respectively. Intriguingly, common polymorphisms in human STN1 associated with longer LTL are also associated with protection CVD-related death in women (Levy *et al*., 2010; Burnett-Hartman *et al*., 2012), providing a link between a protein factor involved in telomere replication, LTL, and aging-associated disorders. The present findings thus raise the possibility that subtle changes that affect Stn1 function or expression levels *in vivo* might be linked to the onset of certain aging-associated disorders.

## Experimental procedures

### Cell culture

BJ cells (ATCC), and derivatives, were cultured in Ham's F10 nutrient mixture (Life Technologies, Grand Island, NY, USA) supplemented with 15% batch-tested fetal bovine serum (Atlanta Biologicals, Lawrenceville, GA, USA), 20 mm L-glutamine (Cellgro, Manassas, VA, USA), 100 U ml^−1^ penicillin, and 100 μg ml^−1^ streptomycin (Cellgro). BJ-hTERT cells were generated by retroviral transduction of BJ cells using the pBabe-hTERT-puro vector followed by drug selection. Cultures were passaged at 1:4 and incubated at 37 °C in atmosphere of 5% CO_2_ and 2% or 21% Oxygen as indicated. Cells were labeled with 1 μg ml^−1^ BrdU (GE Healthcare, Piscataway, NJ, USA), and aphidicolin (Sigma, St. Louis, MO, USA; 0.2 μm) was directly added to the culture medium. Cell proliferation curves were generated by counting cells using a hemocytometer and the formula PD = log2(N_final_/N_initial_), where N_initial_ is the number of cells seeded at each passage and N_final_ is the number of cells recovered from the dish.

### Viral transductions

Retrovirus was generated by calcium phosphate transfection of the Plat-A amphotropic virus packaging cell line (Cell Biolabs, San Diego, CA, USA) and in Phoenix cells. High titer retrovirus was incubated with ∼65% confluent BJ cells for 12 h. Cells were selected with 1 μg ml^−1^ puromycin (Sigma–Aldrich, St Louis, MO, USA) for 48 h.

### ImmunoFISH and Immunofluorescence microscopy

Cultured cells were processed for immunofluorescence analysis as described previously (Herbig *et al*., 2004). Primary antibodies were incubated overnight at 4°C in block buffer (4% BSA in PBST) at indicated concentrations (see below). Following 2 × 5 min washes with PBST, cells were incubated with secondary antibodies as indicated (1:1000 in block buffer) for 1 h at room temperature. Cells were washed 3 × 5 min with PBS, rinsed with water, and mounted using DAPI-containing mounting medium (Vector Laboratories, Burilngame, CA, USA). To detect TIF, fixed cells were dehydrated by sequentially placing them in 70% EtOH, 90% EtOH, and 100% EtOH for 3 min each. After air-drying, nuclear DNA was denatured for 5 min at 80 °C in hybridization buffer containing Cy3-conjugated telomere-specific peptide nucleic acid (PNA; Cy3-(C_3_TA_2_)_3_; Panagene, Korea) at 0.5 μg ml^−1^, 70% formamide, 12 mm Tris-HCl pH = 8.0, 5 mm KCl, 1 mm MgCl_2_, 0.08% Triton X-100, and 0.25% acetylated BSA (Sigma–Aldrich), followed by incubation in the same buffer for 2 h at room temperature. Slides were washed sequentially with 70% formamide/0.6× SSC (90 mm NaCl, 9 mm Na-citrate [pH = 7]; 3 × 15 min), 2XSSC (15 min), PBS (5 min), PBST (5 min), and incubated with block buffer (4% BSA in PBST) for 30 min. Immunostaining using primary polyclonal anti-53BP1 antibodies and secondary AlexaFluor 488-conjugated goat anti-rabbit antibodies (Invitrogen, Carlsbad, CA, USA) was performed as described above. Cells were mounted and analyzed by immunofluorescence microscopy using a Zeiss Axiovert 200 fluorescence microscope, an AxioCamMRm camera (Zeiss), and AxioVision 4.6.3 software (Zeiss, Thornwood, NY). To analyze and quantitate colocalization between telomere signals and 53BP1 foci, images were acquired as z-stacks using a ×100/1.4 oil immersion lens and an ApoTome (Zeiss). ApoTome microscopy eliminates out of focus light and generates shallow focal planes (0.4 mm using a ×100 oil objective). Stacks were merged into a single image using the AxioVision software for easier counting.

### Telomere length measurements by Southern blot

Telomere lengths were measured using Southern blot analysis of the mean of the mean terminal restriction fragments (TRF) lengths, as described (Kimura *et al*., 2010).

### Metaphase telomere analysis

Cells were collected after 48 h in colcemid (final concentration of 1 μg ml^−1^) followed by 10-min incubation in KCl solution (75 mm) at 37 °C and fixed in a freshly prepared 3:1 mix of methanol:glacial acetic acid. Nuclear preparations were dropped onto slides and left to dry overnight at room temperature. FISH was performed as described above using a Cy3-conjugated Cy3-(C3TA2)3 PNA telomeric probe (Panagene, Korea). Chromosomes were counterstained using DAPI.

### Antibodies

The sources and dilutions of antibodies used were as follows: BrdU (Life Technologies), 53BP1 (polyclonal; Novus, Littleton, CO, USA), γH2AX (Upstate, Chicago, IL, USA); α-Tubulin, p53(S15), Chk2(T68), ATR(S428) (Cell Signalling, Danvers, MA, USA); ATM(S1981) (Abcam, Cambridge, MA, USA); p21 (Santa Cruz, Dallas, TX, USA).

### Immunoblotting

Protein extracts were prepared in lysis buffer [20 mm Hepes–KOH, pH 7.9, 0.42 m KCL, 25% glycerol, 0.1 mm EDTA, 5 mm MgCl2, 0.2% NP40, 1 mm DTT, 500 μm PMSF, and 1:100 protease inhibitor cocktail (Sigma–Aldrich)]. 50-μg protein samples were run on 12% SDS-PAGE gels, and proteins were transferred to PVDF membranes (Pall Life Sciences, Pensacola, FL, USA) using a Bio-Rad mini Trans-Blot cell at 350 mA for 90 min. After transfer, membranes were blocked in 5% nonfat dry milk in 1 × TBST (150 mm NaCl, 10 mm Tris-HCl, pH 8.0, 0.05% Tween-20) at room temperature for 1 h and then incubated with primary antibody at 4 °C overnight with gentle agitation. Membranes were washed three times in 1× TBST for 10 min, and subsequently incubated with HRP-conjugated goat anti-mouse or goat anti-rabbit secondary antibodies (Pierce Biotechnology, Rockford, IL, USA) at room temperature for 1 h with shaking. Membranes were washed three times in 1 × TBST for 10 min. Proteins were detected with a SuperSignal West Pico Chemiluminescent Substrate (Pierce Biotechnology), and signals were exposed to Hyblot CL Autoradiography films (Denville, Metuchen, NJ, USA). Autoradiography films were scanned, and band signal intensities were measured by densitometry using the ImageJ (NIH, Bethesda, MD) software (version 1.45).

### Quantitative reverse transcriptase–PCR

RNA was isolated from cells with the Qiagen RNAeasy kit according to the manufacturer's instructions. One microgram of total RNA was subjected to reverse transcription with random hexamer primers using the qScript Flex cDNA Synthesis Kit (Quanta Biosciences, Gaithersburg, MD, USA). Quantitative PCR was performed using an Applied Biosystems real-time PCR detection system with the SYBR Green PCR Master Mix (Life Technologies, Grand Island, NY, USA). The following primers were used: hStn1 forward 5′-CGAGAGATTCATGCCACCG-3′, hStn1 reverse 5′-CGAGAGATTCATGCCACCA-3′; Actin-forward ATAGCACAGCCTGGATAGCAACGTAC, Actin-reverse CACCTTCTACAATGAGCTGCGTGTG. Relative changes in mRNA expression were calculated using actin as an internal reference.

### Senescence-associated β-galactosidase activity

Senescence-associated β-galactosidase activity was analyzed using the SA-βGal Staining Kit according to the manufacturer's instructions (Cell Signaling, Danvers, MA, USA). Briefly, cells were grown on coverslips at a 65% confluency, washed 2 × 3 min in PBS, fixed for 10 min in fixative solution, and stained at 37 °C for 12 h in SA-βGal staining solution at pH 5.5. Subsequently, coverslips were washed 2 × 3 min with PBS and mounted on slides with a mounting medium without DAPI (Vector Laboratories, Burilngame, CA, USA). Cells were analyzed under a light microscope.

### BrdU incorporation assay

Cells were labeled with 1 μg ml^−1^ BrdU (GE Healthcare, Piscataway, NJ, USA; 1:1000). BrdU was added to the culture medium for 48 h. Fixed cells were then stained using the Vectastain ABC Kit (Vector labs, Burlingame, CA, USA), mounted on coverslips and analyzed using a Zeiss Axio Imager A1. Cells that did not incorporate BrdU within the 48-h labeling period were considered senescent.

### Plasmid construction

shRNAs targeting two distinct sequences within the coding region of hSTN1 (Stn1-1: 5′-TGGATCCTGTGTTTCTAGCCT-3′; Stn1-2: 5′-CAGCTTAACCTCACAACTTAA-3′) and within the coding region of GFP (5′-GCAAGCTGACCCTGAAGTTCAT-3′) were subcloned and expressed in pRetroSuper-puro as described (Brummelkamp *et al*., 2002). cDNA of Stn1^A151,C248^ was purchased from OriGene (Rockville, MD, USA). Site-directed mutagenesis was performed using QuikChange Site-Directed Mutagenesis Kit (Agilent Technologies, Santa Clara, CA, USA) to generate cDNA for Stn1^T151,S248^. Both variants were subsequently subcloned and expressed in pBabe-puro.

### Statistical analyses

The observed data were normally distributed and presented as means ± standard deviation (SD). The differences between groups were tested using the unpaired Student's *t-*test or one-way analysis of variance (ANOVA) analysis, followed by Tukey's *post hoc* test for multiple comparisons, as indicated. A linear regression analysis was performed to calculate telomere shortening rates. All *P* values presented are 2-tailed, and a *P *<* *0.05 was chosen for levels of significance. Statistical analyses were performed using spss 16 software package (SPSS, Inc., Chicago, IL, USA) or GraphPad Prism software version 5.0 (San Diego, CA, USA).

## Supporting Information

**Fig. S1** Markers of cellular senescence in hStn1 knockdown cells. **Fig. S2** Knockdown of hStn1 in human BJ fibroblasts activates a DDR but does not enhance the fragile telomere phenotype caused by supraphysiological oxygen tensions. **Fig. S3** Overexpression of hStn1 does not extend replicative lifespan and does not protect cells from the effects of telomeric replication stress. **Fig. S4** Knockdown of hStn1 in hTERT-expressing human cells causes a fragile telomere phenotype that is not further enhanced by DNA polymerase α inhibition.
