# Supplementary material for: Stn1 is critical for telomere maintenance and long-term viability of somatic human cells
Source: Aging Cell. 2015 Feb 14;14(3):372–81. doi: 10.1111/acel.12289 (PMC4406666; doi:10.1111/acel.12289)
Supplement: Supplementary file 1 [file acel0014-0372-sd1.docx]

**Supporting Information**


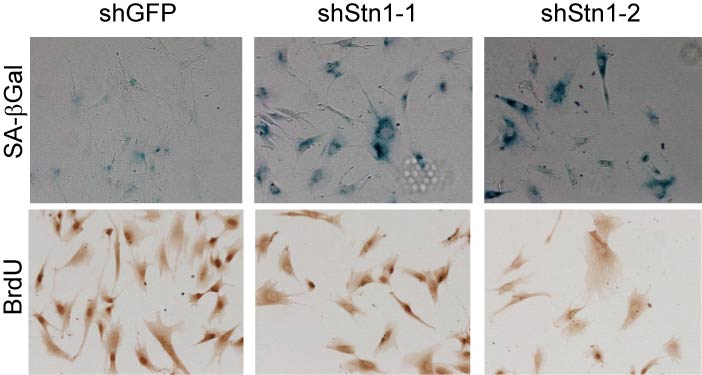


**Figure S1. Markers of cellular senescence in hStn1 knockdown cells**

Representative images of prominent SA-beta-Galactosidase staining and low nuclear BrdU incorporations, added to the culture medium for 48h, in Stn1 knockdown cells, compared to GFP knockdown control cells.


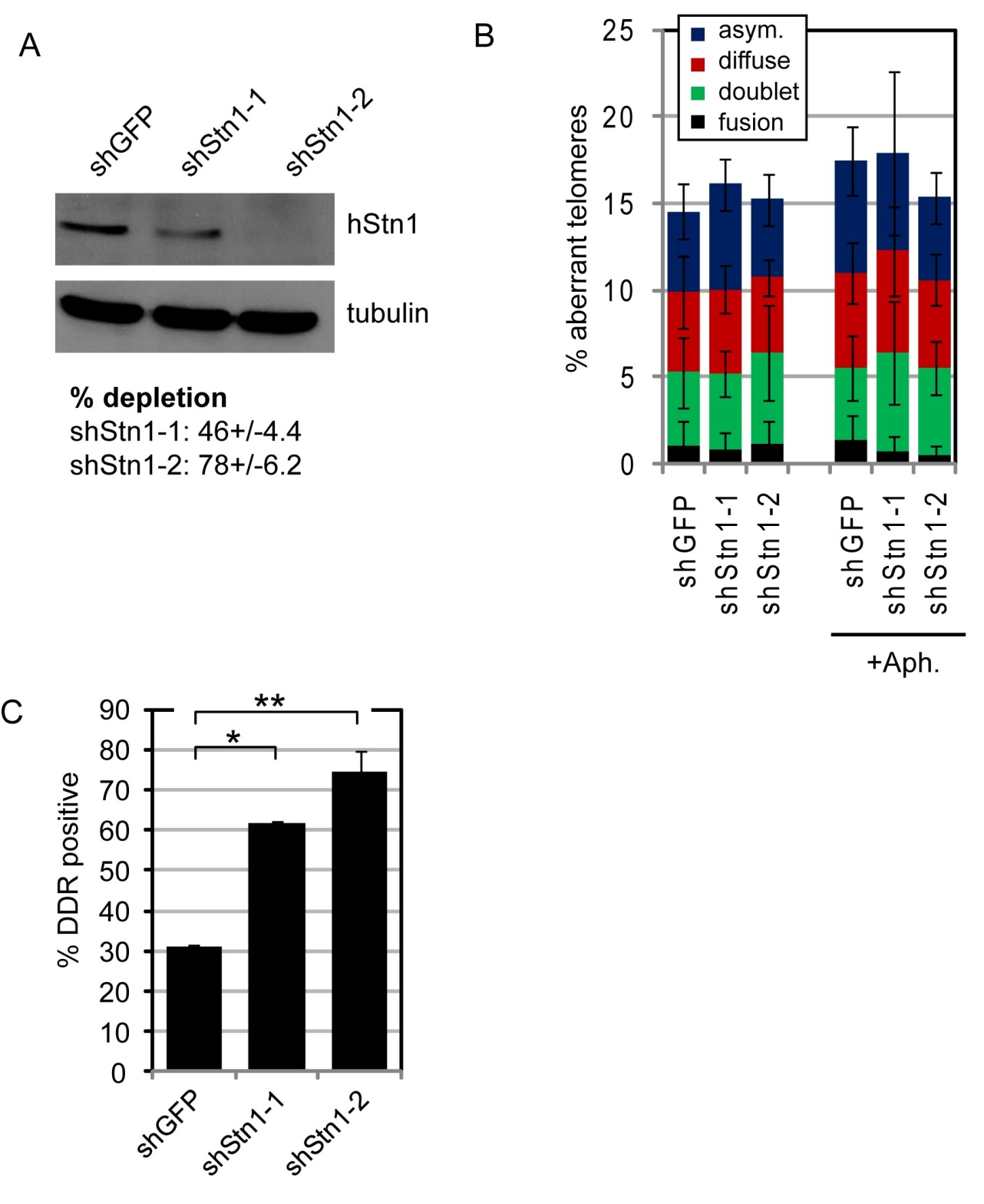


**Figure S2. Knockdown of hStn1 in human BJ fibroblasts activates a DDR but does not enhance the fragile telomere phenotype caused by supraphysiological oxygen tensions.**

**A**: Representative western blot of indicated knockdown cultures grown in atmospheric oxygen tensions (21%). Cells were collected 5 days following retroviral shRNA transduction. Numbers below the western blot indicate the average percent of hStn1 knockdown, compared to GFP control knockdowns, as determined by densitometry. **B:** Quantification of aberrant telomeric structures in indicated knockdown cultures, with or without treatment with 0.2 µM aphidicolin; No significant difference between groups were found. **C:** Quantitation of DDR positive cells, defined as a cell nucleus with one (black bars) or two and greater (Red bars) colocalizations between γH2AX and 53BP1, 3 weeks following transduction of indicated shRNA. *p<0.001 **p=0.007.


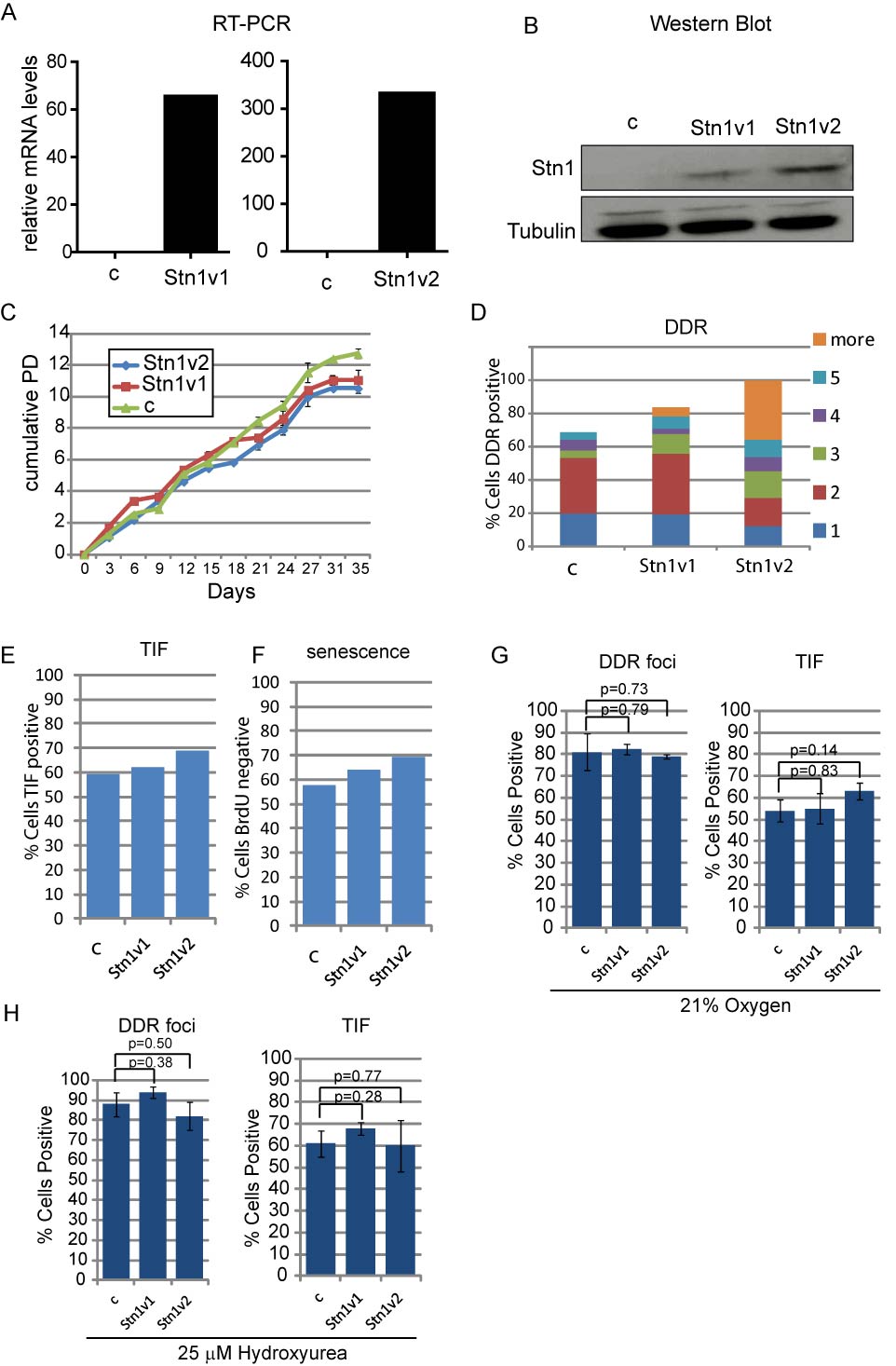


**Figure S3 Overexpression of hStn1 does not extend replicative lifespan and does not protect cells from the effects of telomeric replication stress.**

cDNA’s of two Stn1 variants found in the human population, Stn1^A151,C248^ (Stn1v1) and Stn1^T151,S248^ (Stn1v2), were subcloned into the retroviral vector pBabe-puro. Retrovirally transduced BJ cells were tested for hStn1 overexpression by RT-PCR (**A**) and western blotting (**B**). Tubulin served as a loading control. c: control cells expressing empty vector pBabe-puro. **C**: Proliferation curves of indicated cell cultures. **D**: % of cells displaying 53BP1 foci. Different colors indicate the number of 53BP1 foci per cell nucleus. **E:** % of TIF positive cells. **F:** % of cells that did not incorporate BrdU over a 48h labeling period and were therefore considered senescent. D-E was measured in cultures at day 28. **G**: Left bar graph: % of cells positive for 53BP1 foci as a result of elevated oxygen tensions (21% O2). Right bar graph: % of TIF positive cells as a result of elevated oxygen tensions (21% O2). **H**: Indicated cultures were treated with 25μM hyroxyurea (HU) for 4 days to induce chronic and low levels of DNA replication stress. Left bar graph: % of cells positive for 53BP1 foci as a result of HU treatment. Right bar graph: % cells positive for TIF foci as a result of HU treatment. Note that differences were statistically not significant.


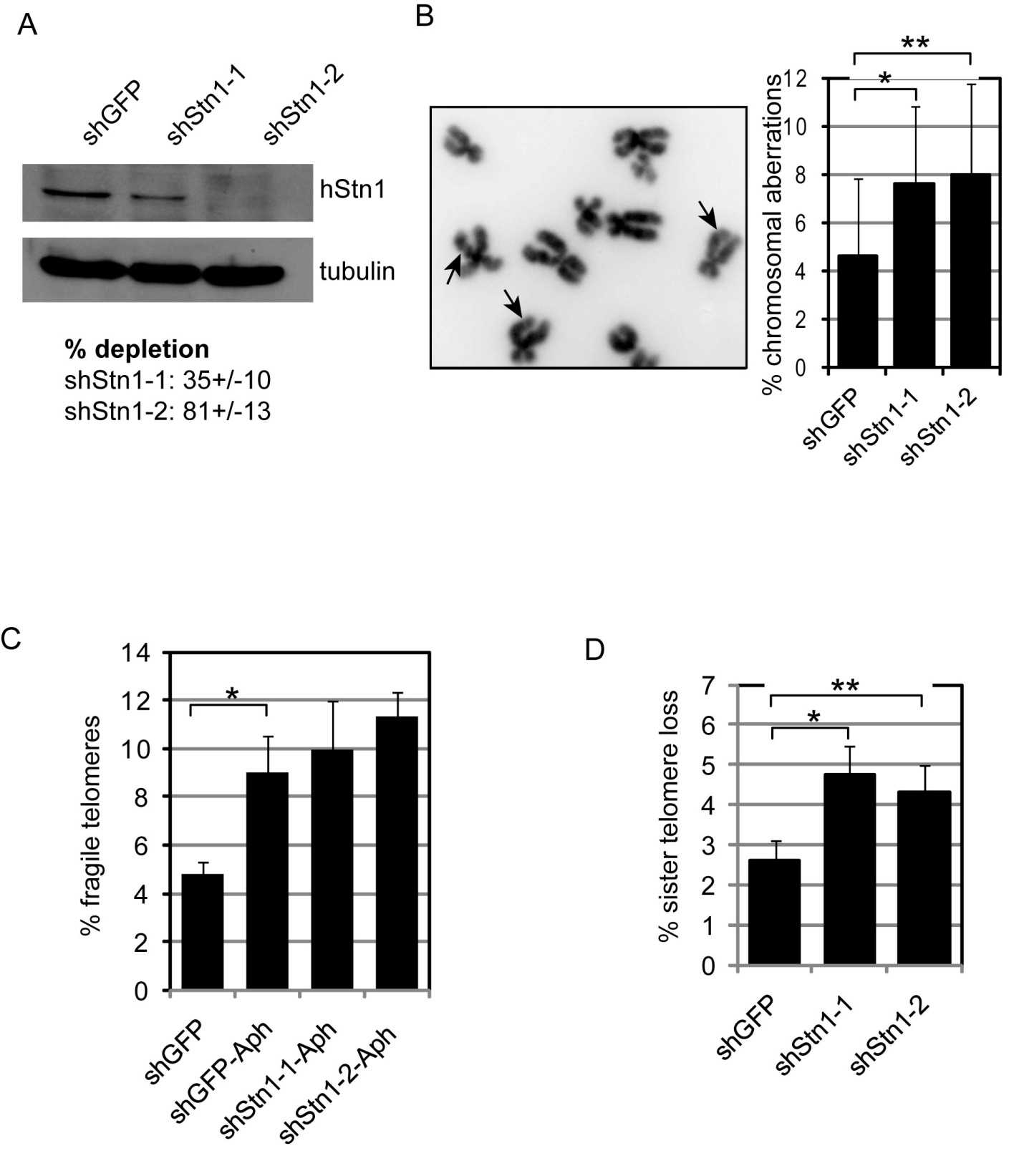


**Figure S4 Knockdown of hStn1 in hTERT expressing human cells causes a fragile telomere phenotype that is not further enhanced by DNA polymerase α inhibition.**

The effect of hStn1 knockdown in telomerase expressing human fibroblast (hTERT) was monitored after the cells had recovered from selection 72h after retroviral **A**: Western blot analysis of hTERT-expressing BJ cells transduced with indicated retroviruses. Cells were collected 5 days after shRNA transduction. Numbers below the image indicate the average percent of hStn1 knockdown as determined by densitometry from three independent experiments. One representative immunoblot is shown. B: Quantitation of chromosomal aberrations from indicated cultures. At least 20 metaphases for each culture were scored. *p=0.0065; **: p= 0.0031. **C:** Quantitation of fragile telomeres in indicated knockdown cultures in the absence or presence of 0.2 µM aphidicolin; * p= 0.017 **D:** Quantitation of the percentage of sister telomere loss; Differences are statistically not significant (*p= 0.07; **p=0.09).
